# Multiple evanescent white dot syndrome in highly myopic eye in which fundus autofluorescence was diagnostically useful: A case report

**DOI:** 10.1097/MD.0000000000032713

**Published:** 2023-02-03

**Authors:** Sumine Harada, Kumiko Kato, Yoshitsugu Matsui, Masahiko Sugimoto, Hisashi Matsubara, Mineo Kondo

**Affiliations:** a Department of Ophthalmology, Mie University Graduate School of Medicine, Tsu, Japan.

**Keywords:** fundus autofluorescence, Multiple evanescent white dot syndrome, optical coherence tomography

## Abstract

Multiple evanescent white dot syndrome (MEWDS) is characterized by multiple white dots in the posterior pole of the eye, but its diagnosis can be difficult in cases when the white dots are not visible. We report an atypical case of MEWDS that developed in pathologic myopia.

**Patient concern:**

A 42-year-old woman presented with floater and reduced and distorted vision in the right eye that she noted 3 days earlier.

**Diagnosis:**

The right eye was highly myopic at -17.0 diopters, and her decimal best-corrected visual acuity was reduced to 0.2 (20/100). Fundus examinations revealed no abnormalities other than myopic fundus changes and previous laser photocoagulation spots. However, fundus autofluorescence (FAF) showed multiple hyperfluorescent dots, and optical coherence tomography showed a high reflective lesion on the retinal pigmental epithelium at the fovea. Adjustments of the brightness and contrast of the conventional fundus images revealed white dots in the same location as the hyperfluorescent spots seen in the FAF images. We diagnosed her with MEWDS.

**Interventions:**

We treated her with systemic administration of 20 mg prednisolone and the dose of prednisolone was reduced by 5 mg every 4 weeks.

**Outcomes:**

The optical coherence tomography and FAF findings gradually normalized, and 5 months later, her decimal visual acuity was restored to 1.0 (20/20).

**Lessons:**

It was suggested that white dots typical to MEWDS may not be evident in pathologic myopia, and FAF images and the brightness and contrast adjustment of fundus images were useful in the diagnosis of atypical MEWDS.

## 1. Introduction

Multiple evanescent white dot syndrome (MEWDS) is a relatively rare retinochoroidal disorder that was first reported by Jampol et al in 1984.^[1]^ MEWDS develops in one eye of healthy young women and is manifested as a sudden decrease of the visual acuity, visual field loss, and photopsia.^[1–3]^ The prognosis of MEWDS is good, and the visual acuity and visual field defects usually recover to almost normal without any treatment.^[1,4]^ The cause of MEWDS has not been definitively determined however a viral infection and related inflammation or autoimmunity may be associated with its pathogenesis because patients often have cold-like symptoms before the onset.^[5,6]^

The main fundus findings of MEWDS is the numerous white dots in the deep retina. However, because these white dots can disappear spontaneously within a few months, those patients with a long interval between the disease onset and the clinical examination may present without the white dots. In these patients, fundus autofluorescence (FAF) has been reported to be helpful in their diagnosis.^[7]^

We report our findings in a young woman who presented with a sudden reduction of vision, and the absence of white dots in the ophthalmoscopic examinations. However, numerous hyperfluorescent dots were detected in the FAF images. After extensive examinations including FAF, she was diagnosed with MEWDS.

## 2. Case report

A 42-year-old woman was examined in a local eye clinic complaining of sudden decrease in her vision, floaters, and distorted vision in her right eye that begun 3 days earlier. She was referred to the Mie University Hospital for further examination and treatment for the unexplained vision reduction. She had undergone laser treatment and scleral buckling surgery for lattice degeneration and retinal detachment in both eyes during her teens and twenties. Both eyes were diagnosed with high myopia and were corrected with hard contact lenses, and her decimal best-corrected visual acuity (BCVA) was 1.0 for the right eye and 0.3 for the left eye.

**Figure 1.**
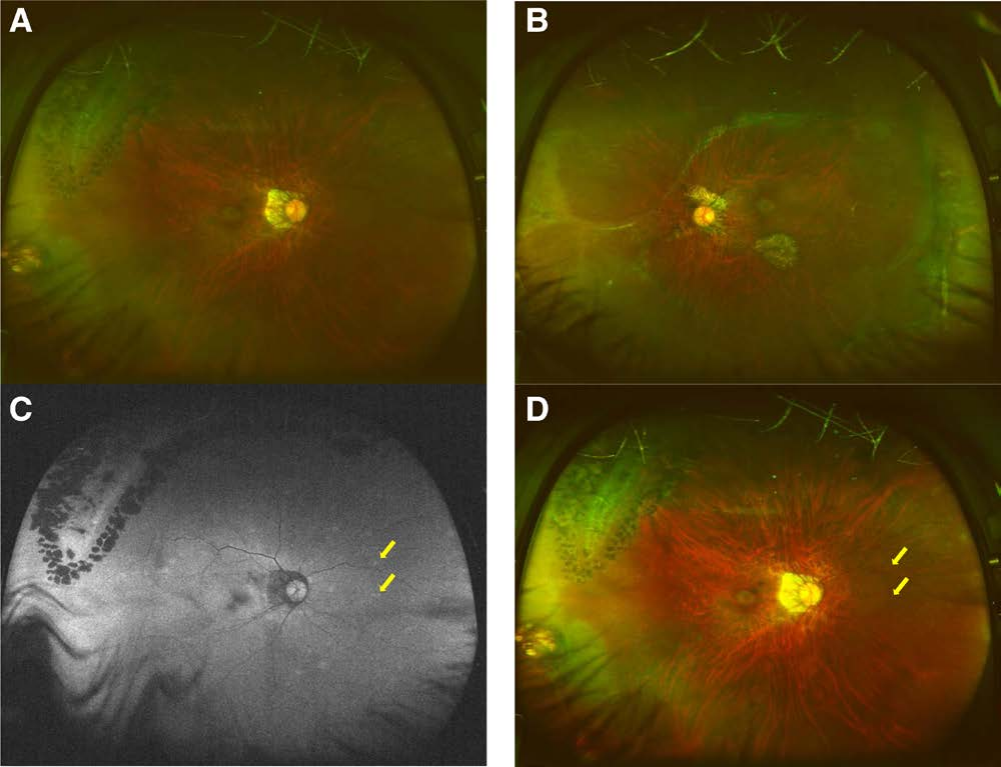
Fundus photographs and fundus autofluorescence (FAF) images taken with the ultra-wide field imaging system at initial examination. (A) Fundus photograph of the right eye showing lattice degeneration, prior laser photocoagulation spots, and the severe peripapillary atrophy around the optic nerve head. (B) Fundus photograph of the left eye showing chorioretinal atrophic lesions caused by the previous surgery and the severe peripapillary atrophy around the optic nerve head. (C) FAF image of the right eye. Numerous hyperfluorescent dots can be seen. (D) Adjusted image of (A) with increased brightness by 20% and increased contrast by 40% reveals white dots (yellow arrows) located at same position as the hyperfluorescent dots in the FAF image (C).

**Figure 2.**
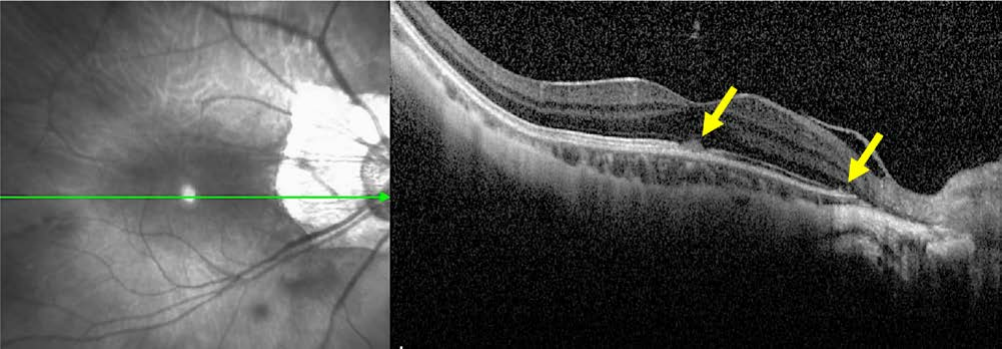
OCT finding at the initial examination showing the highly reflective lesion on the retinal pigmentary epithelium at the fovea and near the optic nerve head (yellow arrows).

**Figure 3.**
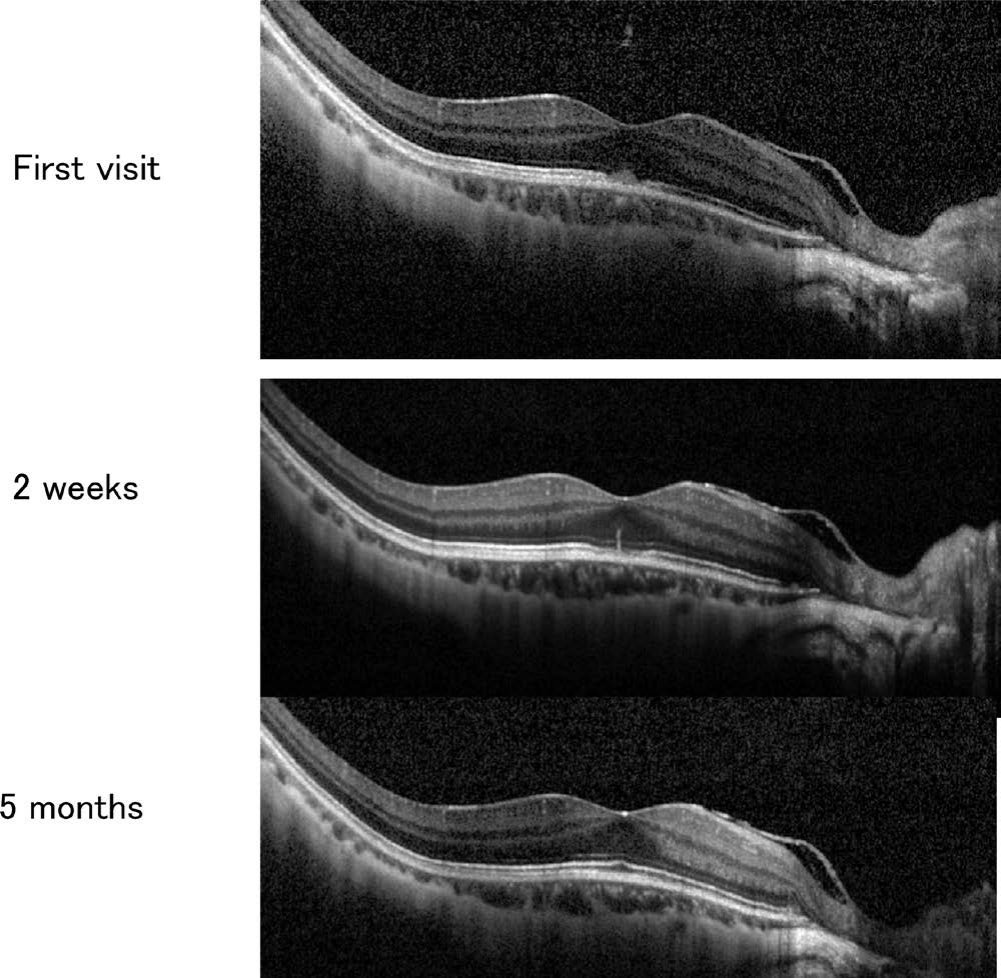
Chronological changes in the OCT findings of the right eye. Upper: At the initial visit, there was a high reflective lesion on the retinal pigmentary epithelium with an interruption of the ellipsoid zone coinciding with the location of the lesion. Middle: 2 weeks after the first visit, the highly reflective lesion has shrunk and integrity of the ellipsoid zone has improved. Lower: 5 months after the first visit, the highly reflective lesion is completely absent and a foveal bulge can be seen. The structure of the ellipsoid zone near the optic nerve head has been normalized.

At her initial examination, her decimal BCVA was 0.2 with a correction of −17.00 DS = −0.50 DC Ax 115° in the right eye and 0.3 with −11.50 DS = −1.00 DC Ax 65° in the left eye. Slit-lamp examination showed mild posterior subcapsular opacities in both eyes, but no obvious inflammation in the anterior segment of the eyes. Fundus examination showed retinal choroidal atrophy in both eyes and scars from the previous surgeries including laser photocoagulation, but there were no obvious abnormalities (Fig. 1A and B). Optical coherence tomography (OCT, Spectralis; Heidelberg, German) showed an interruption of the ellipsoid zone in the central fovea of the right eye with a small highly reflective elevation over the retinal pigment epithelium (Fig. 2). FAF images taken with ultra-wide field (UWF) retinal imaging system (Optos 200Tx, Optos PLC. Dunfermline, UK) had numerous pale hyperfluorescent dots in the right eye (Fig. 1C). We adjusted the brightness and contrast of the fundus images taken with UWF imaging system and found that white dots were present in the same location as the hyperfluorescent dots seen in the FAF images (Fig. 1D). Suspecting choroidal neovascularization or MEWDS associated with high myopia, optical coherence tomography angiography was performed, but no blood flow component was observed in the area of high reflective elevation seen in the OCT images. Fluorescein angiography showed no obvious fluorescent dye leakage consistent with a lesion in the central fovea. Based on the subjective symptoms, fundus images, FAF, and OCT findings, we strongly suspected the patient had MEWDS. As the disease was in her dominant eye, systemic administration of 20 mg prednisolone was started with the expectation of early visual recovery. Two weeks later, the symptoms had improved and the high reflective elevated lesion seen in the OCT images had shrunk (Fig. 3 middle panel). Her decimal BCVA in the right eye recovered to 0.5 after 1 month of treatment, and the hyperfluorescent dots seen in the FAF images at the initial visit had disappeared. The dose of prednisolone was reduced by 5 mg every 4 weeks, and the BCVA of the right eye improved to 0.7 at 2 months, and 1.0 at 5 months of the treatment. The OCT images at 5 months of the treatment showed a complete disappeared of the highly reflective lesion and the foveal bulge (Fig. 3, lower panel).

## 3. Discussion

We have examined a case of atypical MEWDS in which white dots were not evident in the fundus examinations or fundus photographs. However, we suspected MEWDS based on OCT and FAF findings and subjective symptoms. Then, when the contrast and brightness of the fundus photographs were adjusted, white dots became apparent. These white dots were located where the hyperfluorescent dots were located in the FAF images. Thus, the eye was diagnosed as having MEWDS, and the contrast adjustments of the fundus images were helpful in the diagnosis.

The most important finding for the diagnosis of MEWDS is the large number of white dots in the retina. However, it has been reported that the white dots can fade and not be visible in cases when several months have passed since the onset of the disease. It was recently reported that typical MEWDS with white dots showed hyperfluorescent dots in the FAF images. The locations of these hyperfluorescent dots were found to correspond with white dots in the fundus images.^[8–12]^ Thus, it was concluded that FAF can be helpful in the diagnosis of MEWDS even in cases without the white dots in the conventional fundus images.^[8,11]^

In our case, the white dots in the fundus were not evident in spite of the fact that the patient had visited our clinic only 5 days after the onset of the symptoms. We questioned why the typical white dots were not evident in our case? We suggest that 2 factors were involved; performance limitation of the UWF fundus camera, and alterations in the retinal color tone due to the high myopia.

The UWF fundus camera is an advanced device that can record approximately 80% of the fundus in a single shot. However, it is susceptible to opacities of crystalline lens and vitreous, and the images have a slightly greenish tint and is generally darker than the original retinal color tone because it uses 2 different laser beams with different wavelengths.^[13]^ Another relevant study reported that when UWF imaging system was used to screen for diabetic retinopathy, it failed to detect the white lesions in about 60% and small lesions in about 30% of the eyes.^[14]^ Based on these studies, we believe that white dots characteristic for typical MEWDS might be obscured because of the greenish color tone of the images taken with the UWF retinal imaging system.

Most MEWDS in Japan are reported to occur in myopic eyes of -5.30 ± 4.58 diopters,^[15]^ and there are few reports of MEWDS occurring in eyes with pathological myopia as in this case. A case report of MEWDS occurring in high myopia with a refractive error of -14 D and retinal choroidal atrophy reported that the white dots on the fundus were unclear and FAF was useful for diagnosis.^[16]^ These studies have suggested that it is not easy to detect the white dots characteristic to MEWDS in eyes with high myopia.

In our case, the patient had very high myopia and posterior subcapsular opacities of the lens, and the low quality of the fundus photograph made it difficult to detect the white dots. However, by adjusting the brightness and contrast of the fundus images, the white dots became apparent. When making a diagnosis using images taken with an ultra-wide field fundus camera, we should consider the possibility that some lesions may not be clearly visible depending on their size and color tone.

The OCT examinations of MEWDS patients have shown that the ellipsoid zone is disrupted and a dome-shaped high reflective material is present on the retinal pigment epithelium or retinal outer granular layer.^[12,17]^ The mechanism causing the highly reflective material is unknown, but it has been suggested that it may be residues of the outer segments of the photoreceptors.

According to the reports by the SUN Working Group in 2021, OCT image processing technology using deep learning can differentiate MEWDS from variety of posterior uveitis cases with more than 90% accuracy.^[18]^ Thus, these OCT findings were also considered useful for the diagnosis of MEWDS in cases such as the present case in which the typical white dots were not seen.

There were at least 2 limitations. First, there have been reported that indocyanine green angiography is also useful for diagnosis of MEWDS, but we did not perform indocyanine green angiography in this patient. Second, we have treated our patient with systemic steroids. A clinical course of spontaneous resolution without the use of steroids would have provided strong evidence that this patient had MEWDS.

In conclusion, we have studied a case of atypical MEWDS without the pathognomonic white dots in the fundus. When MEWDS occurs in high myopia, or the examination occurs a long interval since the disease onset, the white dots typical to MEWDS may be obscured or absent. In such cases, every effort should be made to make a diagnosis by the use of FAF and OCT findings and contrast adjustment of fundus photographs.

## Acknowledgments

We thank Professor Emeritus Duco I. Hamasaki of the Bascom Palmer Eye Institute of the University of Miami (Miami, FL, USA) for critical discussion and final manuscript revisions.

## Author contributions

SH, KK, and MK wrote the main manuscript. YM, MS, and HM collected the data. All authors reviewed the manuscript.

**Conceptualization:** Kumiko Kato, Mineo Kondo.

**Data curation:** Mineo Kondo, Hisashi Matsubara, Masahiko Sugimoto.

**Writing—original draft:** Sumine Harada.

**Writing—review and editing:** Kumiko Kato, Hisashi Matsubara, Masahiko Sugimoto, Yoshitsugu Matsui, Mineo Kondo.
